# An avoidance trap? Within-person dynamics between social interaction anxiety and the tendency to avoid physical activity and sport among Chinese university students: a four-wave random-intercept cross-lagged panel study

**DOI:** 10.3389/fpsyg.2026.1914632

**Published:** 2026-08-21

**Authors:** Dongyu Liu, Xianlin Xiao, Rui Tang

**Affiliations:** 1 School of Physical Education, Southwest Petroleum University, Chengdu, Sichuan, China; 2 Faculty of Management, Shinawatra University, Pathum Thani, Thailand; 3 College of Sports and Great Health, Sichuan Technology and Business University, Meishan City, Sichuan, China

**Keywords:** exercise avoidance, physical activity and sport, random-intercept cross-lagged panel model, social interaction anxiety, university students, within-person dynamics

## Abstract

Physical activity and sport settings may become socially threatening when students anticipate negative evaluation of their bodies, competence, or behavior. Yet it remains unclear whether social interaction anxiety precedes exercise-related avoidance and whether such avoidance, in turn, precedes later anxiety. This study examined reciprocal longitudinal associations at both the between- and within-person levels between social interaction anxiety (SIA) and the tendency to avoid physical activity and sport (TAPAS), assessed with a 10-item self-report scale. A baseline cohort of 1,676 university students aged 18 years or older from nine universities in Sichuan Province, China, completed four assessments approximately 12 weeks apart during the 2024–2025 academic year. A random-intercept cross-lagged panel model separated stable between-person differences from time-specific within-person deviations, and longitudinal scalar invariance was supported for both measures. At the between-person level, higher average SIA was associated with higher average TAPAS (*r* = 0.381, *p* < 0.001). At the within-person level, elevations in SIA relative to students’ usual levels predicted subsequent elevations in TAPAS (βs = 0.121–0.127), and elevations in TAPAS predicted subsequent elevations in SIA (βs = 0.126–0.133); all cross-lagged effects were significant at *p* < 0.001. Both constructs also showed significant within-person temporal continuity. The reciprocal pattern was stable across the three approximately 12-week intervals and remained substantively unchanged after adjustment for baseline sex, year of study, pre-university residence, and body mass index category. The findings are consistent with a reciprocal avoidance process across an academic year and suggest that campus mental-health and physical-activity initiatives may benefit from jointly addressing social-evaluative concerns and barriers embedded in exercise settings. Because the study was observational and relied on self-report measures, the associations should not be interpreted causally.

## 1 Introduction

Physical activity and sport are often promoted as resources for university students’ mental health. Yet gyms, physical education classes, fitness programs, and team sports also make bodies and physical competence visible to peers. For students who fear scrutiny, these settings may function as social-evaluative environments. Social interaction anxiety (SIA) refers to anxiety, tension, and discomfort during interpersonal encounters, particularly when unfavorable evaluation is anticipated. In the present study, SIA is treated as a dimensional symptom domain assessed continuously; scores on the 10-item Social Interaction Anxiety Scale (SIAS-10) do not establish a clinical diagnosis of social anxiety disorder. A meta-analysis estimated that 23.5% of Chinese children, adolescents, and young adults experienced elevated social anxiety symptoms, although prevalence varied across measures and studies (Tang et al., 2022). University life combines dense peer-facing demands with exercise settings such as compulsory physical education, campus recreation, and shared fitness facilities. Greater autonomy also allows students to withdraw from many of these settings. These features make university contexts especially informative for examining how social-evaluative concerns become exercise-related avoidance (Auerbach et al., 2018; Lipson et al., 2022; Morrison and Heimberg, 2013).

The tendency to avoid physical activity and sport (TAPAS) captures an inclination to avoid such settings because of weight stigma, appearance concerns, perceived physical ability, or anticipated negative evaluation (Bevan et al., 2022). TAPAS differs from actual physical activity level. Low activity may result from injury, time constraints, or limited access, whereas a student can experience strong avoidance motivation even while attending mandatory physical education. TAPAS therefore represents an evaluative and motivational barrier rather than the amount of activity performed. The original 10-item scale showed a unidimensional structure and associations with lower enjoyment of and participation in physical activity and sport (Bevan et al., 2022). The Mandarin Chinese 10-item version demonstrated a one-factor structure, convergent and discriminant validity, and measurement invariance across sex, physical activity level, and weight status among mainland Chinese university students (Saffari et al., 2023). A subsequent Rasch study supported the scale’s overall functioning but identified misfit for Item 10 and reported an acceptable nine-item solution (Fan et al., 2023). Psychometric evidence has also been extended to Italian and Turkish samples, with the Turkish adaptation retaining nine items (Korkmaz et al., 2026; Soraci et al., 2024).

Cognitive-behavioral models provide a basis for expecting SIA to precede TAPAS. Socially anxious individuals tend to overestimate the likelihood and consequences of negative evaluation, focus attention on internal sensations and perceived appearance, and form negatively biased impressions of how they appear to others (Hofmann, 2007; Rapee and Heimberg, 1997). Exercise settings can activate these processes because physique, coordination, competence, sweating, and interactions with unfamiliar peers may all be observable. A temporary elevation in SIA may therefore increase the perceived threat of exercise-related situations and strengthen later avoidance. Cross-sectional evidence is consistent with this pathway: social anxiety has been associated with exercise-avoidance motivation and lower self-reported exercise among undergraduates, and a recent study of Chinese university students linked social anxiety with greater exercise avoidance after demographic adjustment (Horenstein et al., 2021; Li et al., 2025). These studies cannot determine whether changes in SIA precede later changes in TAPAS within the same student.

The anxiety-driven pathway leaves open whether exercise-related avoidance may feed back into broader social anxiety. Avoidance can produce immediate relief, which negatively reinforces withdrawal, while also limiting opportunities to discover that feared outcomes may not occur or may be less consequential than expected. Safety behaviors can similarly restrict corrective learning when benign outcomes are attributed to protective strategies rather than to the relative safety of the situation (Hofmann, 2007; Piccirillo et al., 2016). Applied to physical activity and sport, avoidance may remove opportunities to tolerate visibility, receive benign feedback, develop physical mastery, and interact informally with peers. Repeated withdrawal from these settings could therefore preserve concerns about scrutiny or allow them to generalize beyond exercise contexts. Evidence that physical activity interventions may reduce social anxiety offers indirect support, although the intervention literature remains limited and does not establish the proposed mechanism (Zika and Becker, 2021).

Together, these pathways imply a potentially reciprocal process. The term avoidance trap is used here as a heuristic label for a hypothesized cycle grounded in negative reinforcement and restricted corrective learning; it is not presented as an established diagnosis or formal theory. Direct evidence remains scarce. Studies connecting social anxiety with exercise avoidance have largely been cross-sectional (Horenstein et al., 2021; Li et al., 2025), while longitudinal work has generally assessed exercise participation rather than psychologically motivated avoidance. A recent four-wave RI-CLPM among Chinese junior high school students examined physical exercise, body image, and social anxiety, but it involved younger adolescents and did not isolate the reciprocal relation between interaction anxiety and TAPAS (Xing et al., 2026). Depressive symptoms, body dissatisfaction, self-esteem, perceived social support, and academic stress may also contribute to temporal coupling between SIA and TAPAS; these mechanisms were not directly tested here.

Testing the reciprocal proposition requires separating stable differences between students from fluctuations occurring within the same student. A positive association between students may reflect enduring body-image concerns, personality, or long-term experiences of stigma without showing that a rise in SIA within a particular student is followed by greater avoidance. Conventional cross-lagged panel models can combine these sources of variation (Hamaker et al., 2015). The random-intercept cross-lagged panel model (RI-CLPM) separates stable between-person components from time-specific within-person deviations, permitting a direct test of whether an elevation in one construct relative to a student’s usual level predicts a later elevation in the other (Hamaker et al., 2015; Mulder and Hamaker, 2021). Four waves provide three adjacent intervals across which the consistency of the prospective associations can be evaluated. The observational RI-CLPM strengthens temporal interpretation but does not establish causality (Berry and Willoughby, 2017; Rohrer and Murayama, 2023).

**FIGURE 1:**
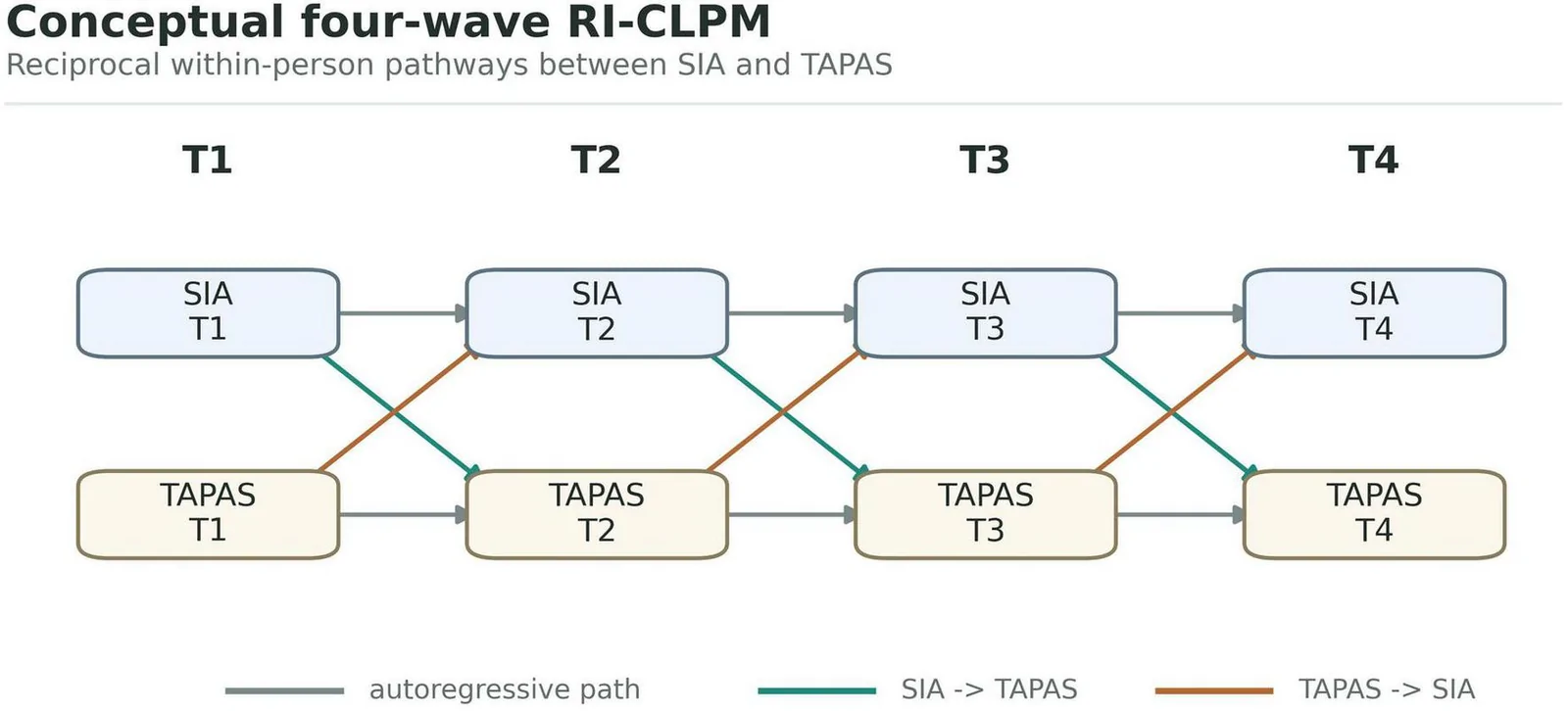
Hypothesized reciprocal within-person relations between social interaction anxiety and the tendency to avoid physical activity and sport across four waves. SIA = social interaction anxiety; TAPAS = tendency to avoid physical activity and sport; T1–T4 = Time 1–Time 4. Horizontal arrows represent within-construct autoregressive paths, and diagonal arrows represent reciprocal cross-lagged paths. The Figure depicts the within-person component of the hypothesized random-intercept cross-lagged panel model. Random intercepts, the covariance between the random intercepts, and within-wave covariances between the within-person components are omitted for visual clarity.

The study examined reciprocal longitudinal associations between SIA and TAPAS at both levels. H1 predicted that higher person-level mean SIA would be associated with higher person-level mean TAPAS. H2 predicted that within-person elevations in SIA would precede higher TAPAS at the subsequent wave. H3 predicted that within-person elevations in TAPAS would precede higher SIA at the subsequent wave. Autoregressive paths were estimated for both constructs, and the two directional pathways were modeled simultaneously across all three approximately 12-week intervals. Figure 1 presents the hypothesized within-person model.

## 2 Materials and methods

### 2.1 Participants and procedure

This four-wave, multi-institutional prospective panel study recruited university students through classroom-based convenience sampling at nine universities in Sichuan Province, China: Xihua University, Southwest Petroleum University, Chengdu Technological University, Sichuan University, University of Electronic Science and Technology of China, Southwestern University of Finance and Economics, Southwest Jiaotong University, Civil Aviation Flight University of China, and Sichuan University of Science and Engineering. Students were eligible when they were enrolled at a participating university at baseline, were aged 18 years or older, could complete the questionnaire in Mandarin Chinese, and agreed to longitudinal follow-up. The study was approved by the Ethics Committee of the School of Physical Education, Southwest Petroleum University. Written informed consent was obtained before participation, and no compensation was provided. Unique study codes linked responses across waves without using names or student identification numbers in the analytic data.

At T1, 1,913 paper-and-pencil questionnaires were distributed and 1,763 were returned, giving a questionnaire return rate of 92.16%. After screening, 87 questionnaires were excluded because consent was absent, age was reported as younger than 18 years, a longitudinal code indicated a duplicate submission, valid baseline scores could not be calculated for both focal measures, or the prespecified instructed-response item was answered incorrectly. The instructed-response item directed respondents to select a specified response option (1 or 5). The baseline analytic cohort comprised 1,676 participants, corresponding to 95.07% of returned questionnaires and 87.61% of all questionnaires distributed. No information was collected from students who declined participation or did not return a questionnaire, so respondents and nonrespondents could not be compared.

The same questionnaire format, item wording, response options, and standardized instructions were used at all waves. Surveys were administered in classrooms or other quiet teaching rooms in September and December 2024 and March and June 2025, at intervals of approximately 12 weeks. The T1–T2 and T3–T4 intervals occurred mainly within teaching semesters, whereas T2–T3 crossed the winter recess. The cohort was closed after T1; no replacement participants were added, and students who missed one wave remained eligible to return later. The lagged coefficients are therefore interpreted as approximately 12-week associations across one academic year (Dormann and Griffin, 2015; Kuiper and Ryan, 2018).

Item-level and wave-level missingness were handled separately. A prespecified study-specific rule required at least eight valid responses for each 10-item scale. Scores based on eight or nine items were prorated by multiplying the available-item mean by 10, limiting proration to cases with no more than 20% item nonresponse. Scores with fewer than eight valid items were coded missing, and the instructed-response item was not included in either scale. A complete-item sensitivity analysis required all 10 responses. At the longitudinal-model stage, full information maximum likelihood (FIML) used all available scale scores from the 1,676 baseline participants.

**FIGURE 2:**
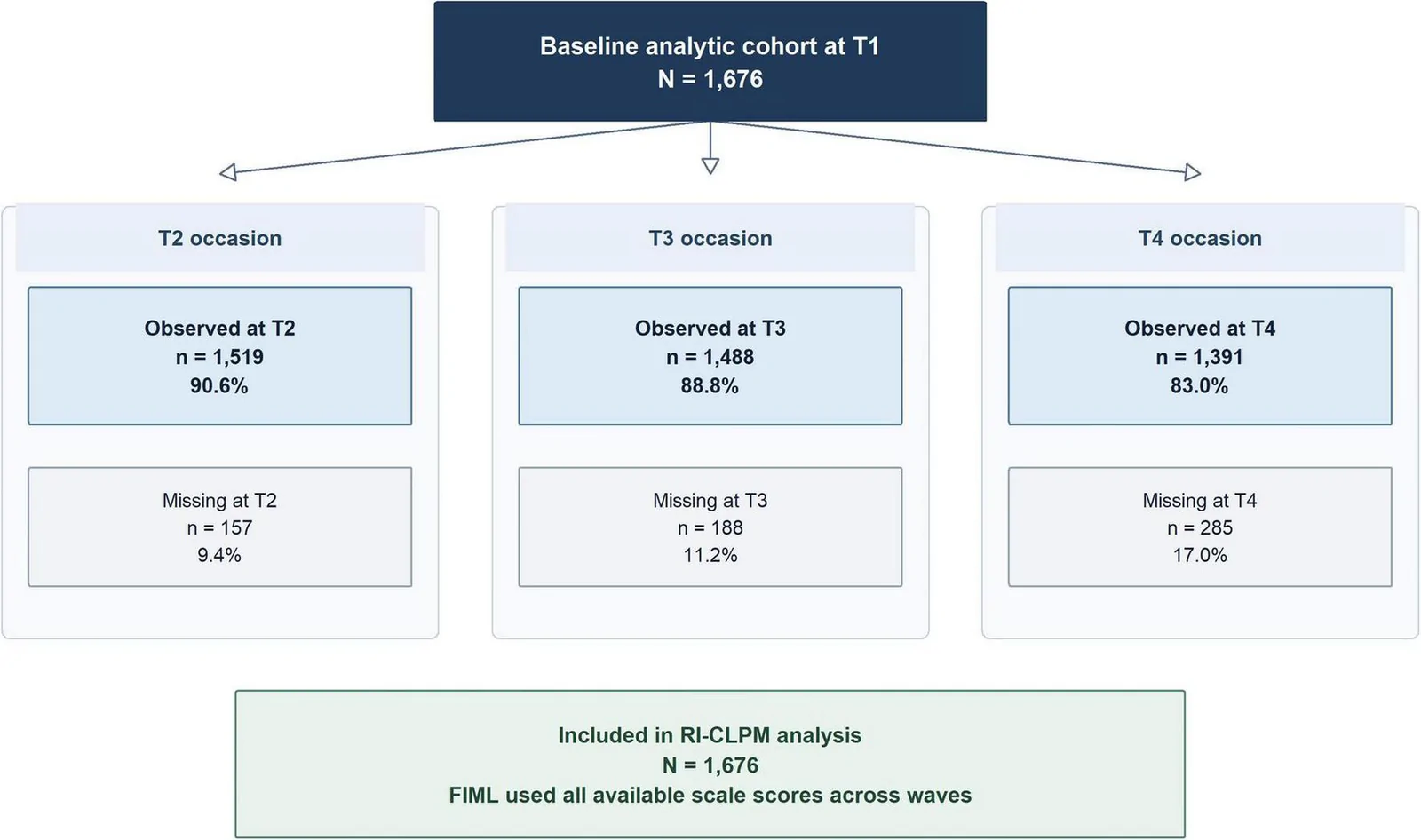
Occasion-specific observation and wave-level missingness across the four-wave study. RI-CLPM = random-intercept cross-lagged panel model; FIML = full information maximum likelihood. Percentages were calculated relative to the T1 analytic cohort (N = 1,676). T2, T3, and T4 represent occasion-specific observations rather than a monotonic retention sequence. Participants missing at one wave remained eligible to participate at subsequent waves, and the wave-specific missing groups could therefore overlap. All 1,676 baseline participants were included in the RI-CLPM, with FIML using the scale data available at each wave.

Participant flow is shown in Figure 2. Observation was defined separately at each follow-up relative to the T1 cohort: 1,519 students were observed at T2, 1,488 at T3, and 1,391 at T4. The corresponding wave-specific missing groups could overlap because students were allowed to return after an earlier absence. All baseline participants remained in the RI-CLPM analytic sample, with FIML using the information available at each occasion.

### 2.2 Measurements

At every wave, participants completed the same Mandarin Chinese self-report instruments. McDonald’s omega was treated as the preferred reliability estimate, while Cronbach’s alpha was retained to facilitate comparison with previous validation studies (McNeish, 2018).

SIA was assessed with the Chinese 10-item Social Interaction Anxiety Scale (SIAS-10). The abbreviated form was selected to reduce respondent burden across four repeated assessments while retaining a validated unidimensional measure of interaction anxiety in Chinese university students (Kupper and Denollet, 2012; Song et al., 2024). Items are rated from 0 (not at all characteristic or true of me) to 4 (extremely characteristic or true of me), with all items keyed in the symptom direction. Total scores range from 0 to 40; higher scores indicate greater SIA.

TAPAS was assessed with the Mandarin Chinese 10-item Tendency to Avoid Physical Activity and Sport Scale (Bevan et al., 2022; Saffari et al., 2023). Items refer to related weight-, appearance-, competence-, and evaluation-based reasons for avoiding physical activity or sport and are treated as indicators of a single avoidance construct rather than separate subscales. Responses range from 1 (strongly disagree) to 5 (strongly agree), yielding total scores from 10 to 50; higher scores indicate stronger avoidance tendency.

Baseline covariates included sex, year of study, pre-university residence, and body mass index (BMI) category. Height and weight were self-reported, and BMI was calculated as weight in kilograms divided by height in meters squared. Chinese adult criteria classified participants as underweight (<18.5 kg/m^2^), normal weight (18.5–23.9 kg/m^2^), overweight (24.0–27.9 kg/m^2^), or obese (≥28.0 kg/m^2^; National Health and Family Planning Commission of the People’s Republic of China, 2013).

### 2.3 Data analysis

Data management, descriptive statistics, reliability estimation, and attrition analyses were conducted in R 4.5.3; longitudinal measurement models, RI-CLPMs, and Monte Carlo simulations were estimated in Mplus 8.3 (Muthén and Muthén, 1998–2017). Demographic characteristics were summarized with frequencies and percentages; SIA and TAPAS were summarized with means and standard deviations. Reliability was estimated at each wave with alpha and omega, and Pearson correlations were calculated among the eight repeated scale scores. All tests were two-tailed with α = 0.05, and 95% confidence intervals were reported where applicable. FIML handled incomplete scale scores under a missing-at-random (MAR) assumption conditional on variables in the model. The plausibility of this assumption was examined by comparing observed and missing participants at T2, T3, and T4 on baseline SIA, TAPAS, and demographic characteristics and by estimating multivariable logistic models of wave-specific observation. These analyses can identify observed predictors of missingness but cannot verify MAR (Enders and Bandalos, 2001; Graham, 2009).

Longitudinal measurement invariance was examined separately for the SIAS-10 and TAPAS using item-level one-factor confirmatory models. Same-item residuals were allowed to correlate across waves. Configural models were followed by metric models constraining corresponding loadings, scalar models additionally constraining item intercepts, and strict models additionally constraining residual variances. Scalar invariance was sufficient for comparing construct scores and structural relations; strict invariance was evaluated as an additional robustness test.

Model fit was evaluated from the joint pattern of the chi-square statistic, comparative fit index (CFI), Tucker–Lewis index (TLI), root mean square error of approximation (RMSEA) with its 90% confidence interval, and standardized root mean square residual (SRMR). Conventional values were used as descriptive reference points rather than mechanical decision rules. Changes of ΔCFI ≤0.010, ΔRMSEA ≤0.015, and ΔSRMR ≤0.030 for metric or ≤0.010 for scalar and strict comparisons were used to evaluate invariance alongside absolute fit, parameter plausibility, and model complexity (Chen, 2007; Putnick and Bornstein, 2016).

A four-wave RI-CLPM decomposed repeated SIA and TAPAS scores into stable random intercepts and wave-specific within-person components (Hamaker et al., 2015). The random intercepts were allowed to correlate. Adjacent autoregressive and reciprocal cross-lagged paths were estimated among the within-person components, with the T1 within-person covariance and T2–T4 residual covariances freely estimated. Random intercepts were specified as orthogonal to the within-person components. The random-intercept correlation represented stable between-person covariation, whereas lagged paths represented prospective deviations within the same student.

Four nested specifications tested temporal equality of the lagged paths. M1 freely estimated all autoregressive and cross-lagged paths; M2 constrained autoregressive paths across time; M3 constrained each directional cross-lagged path across time; and M4 imposed both sets of constraints. Approximate temporal stability was considered plausible because the waves were equally spaced within one academic year, but it was tested rather than assumed because the T2–T3 interval crossed the winter recess. Equality constraints were imposed on unstandardized paths; standardized coefficients were reported for interpretation and could vary slightly because wave-specific variances differed. The more parsimonious model was retained when constraints did not materially worsen fit.

Sensitivity analyses compared M4 with M1 and estimated a covariate-adjusted M4 in which baseline sex, year of study, pre-university residence, and BMI category predicted both random intercepts. These covariates were selected for substantive relevance to stable differences in social anxiety, body-related concerns, and access to physical activity settings. Two scoring sensitivities were also examined: a nine-item TAPAS model excluding Item 10 (“I would prefer to participate in physical activity in a more private setting”), which had shown Rasch misfit in a mainland Chinese sample (Fan et al., 2023), and a complete-item analysis requiring all 10 responses. Both used the same cohort and RI-CLPM structure as the primary analysis.

To characterize sample-size adequacy without relying on a single population model, a Monte Carlo sensitivity analysis varied standardized cross-lagged effects (0.05, 0.07, 0.10, and 0.12), intraclass correlations (0.30, 0.50, and 0.60), and cumulative T4 attrition (15%, 20%, and 25%), with at least 5,000 replications per directional scenario. The grid covered conservative to moderately small lagged effects and a broad range of between-person variance and attrition assumptions. The analysis evaluated the sensitivity of the sample-size conclusion rather than setting the recruitment target. Full simulation details and the resulting 80% power thresholds are reported in the Supplementary Material (Mulder, 2023).

## 3 Results

### 3.1 Participant characteristics

The baseline analytic sample comprised 1,676 university students aged 18 years or older. The sample was nearly evenly distributed by sex, with 840 men (50.12%) and 836 women (49.88%). Participants aged 19 and 20 years were the two largest age groups, each accounting for 23.93% of the sample. Freshmen and sophomores together represented 56.56% of participants. Slightly more than half reported an urban pre-university residence (52.80%), and 63.07% were classified as having normal weight. Table 1 presents the full baseline characteristics.

**TABLE 1:** Baseline demographic characteristics of the analytic sample.

Characteristic	Category	*n*	%
Sex	Male	840	50.12
	Female	836	49.88
Age	18 years	319	19.03
	19 years	401	23.93
	20 years	401	23.93
	21 years	321	19.15
	≥22 years	234	13.96
Year of study	Freshman	479	28.58
	Sophomore	469	27.98
	Junior	433	25.84
	Senior	295	17.60
Pre-university residence	Urban	885	52.80
	Rural	791	47.20
BMI category	Underweight	139	8.29
	Normal weight	1,057	63.07
	Overweight	336	20.05
	Obesity	144	8.59

*N* = 1,676. BMI, body mass index. Pre-university residence refers to the participant’s primary place of residence before university enrollment. BMI categories were defined as underweight (<18.5 kg/m^2^), normal weight (18.5–23.9 kg/m^2^), overweight (24.0–27.9 kg/m^2^), and obesity (≥28.0 kg/m^2^).

### 3.2 Descriptive statistics, reliability, and bivariate correlations

Descriptive statistics, internal-consistency estimates, and bivariate correlations for social interaction anxiety (SIA) and the tendency to avoid physical activity and sport (TAPAS) across the four waves are presented in Table 2. Mean SIA scores ranged from 17.91 at T1 to 18.85 at T4, whereas mean TAPAS scores ranged from 29.73 at T1 to 30.57 at T4. These values indicated modest descriptive increases in both constructs across the observation period, although no inference regarding mean-level change was based on these descriptive values alone.

**TABLE 2:** Descriptive statistics, internal consistency, and bivariate correlations across four waves.

No.	Variable	*M*	*SD*	α	ω	1	2	3	4	5	6	7	8
1	SIA T1	17.91	8.81	0.904	0.920	–	–	–	–	–	–	–	–
2	SIA T2	18.32	9.09	0.912	0.926	0.742							
3	SIA T3	18.45	8.98	0.906	0.922	0.673	0.744						
4	SIA T4	18.85	9.26	0.912	0.927	0.620	0.686	0.753					
5	TAPAS T1	29.73	8.97	0.910	0.925	0.203	0.274	0.294	0.248				
6	TAPAS T2	29.89	9.03	0.915	0.929	0.265	0.233	0.303	0.274	0.744			
7	TAPAS T3	30.33	9.02	0.912	0.927	0.265	0.291	0.269	0.308	0.662	0.759		
8	TAPAS T4	30.57	8.94	0.912	0.927	0.226	0.276	0.293	0.238	0.613	0.657	0.751	

SIA = social interaction anxiety; TAPAS = tendency to avoid physical activity and sport; T1–T4 = Time 1–Time 4; α = Cronbach’s alpha; ω = McDonald’s omega. SIA scores could range from 0 to 40, and TAPAS scores could range from 10 to 50. All non-diagonal correlations were significant at p<.001.

Both instruments demonstrated consistently high internal reliability. Cronbach’s alpha coefficients ranged from 0.904 to 0.915, and McDonald’s omega coefficients ranged from 0.920 to 0.929. Within-construct correlations across waves were moderate to strong, ranging from 0.620 to 0.753 for SIA and from 0.613 to 0.759 for TAPAS. SIA and TAPAS were positively correlated both concurrently and prospectively, with cross-construct correlations ranging from 0.203 to 0.308. All non-diagonal correlations were statistically significant at *p* < 0.001.

### 3.3 Longitudinal measurement invariance

The configural models fitted well for both constructs, and constraining corresponding loadings and intercepts produced negligible changes in fit (Table 3). Across the metric and scalar comparisons, the absolute change in CFI did not exceed 0.001, the absolute change in RMSEA did not exceed 0.002, and the change in SRMR did not exceed 0.004.

**TABLE 3:** Longitudinal measurement invariance of SIA and TAPAS across four waves.

Variable	Model	χ^2^	*df*	*p*	CFI	TLI	RMSEA [90% CI]	SRMR	ΔCFI	ΔRMSEA	ΔSRMR
SIA	Configural	701.805	674	0.222	0.999	0.999	0.005 [0.000, 0.009]	0.015	–	–	–
	Metric	735.435	704	0.200	0.999	0.999	0.005 [0.000, 0.009]	0.019	0.000	0.000	0.004
	Scalar	767.812	731	0.167	0.999	0.999	0.005 [0.000, 0.009]	0.020	0.000	0.000	0.001
	Strict	801.603	761	0.149	0.999	0.999	0.006 [0.000, 0.009]	0.021	0.000	0.001	0.001
TAPAS	Configural	700.488	674	0.233	0.999	0.999	0.005 [0.000, 0.009]	0.015	–	–	–
	Metric	715.349	704	0.375	1.000	1.000	0.003 [0.000, 0.008]	0.017	0.001	-0.002	0.002
	Scalar	741.057	731	0.390	1.000	1.000	0.003 [0.000, 0.008]	0.017	0.000	0.000	0.000
	Strict	764.696	761	0.456	1.000	1.000	0.002 [0.000, 0.008]	0.018	0.000	-0.001	0.001

SIA, social interaction anxiety; TAPAS, tendency to avoid physical activity and sport; CFI, comparative fit index; TLI, Tucker–Lewis index; RMSEA, root mean square error of approximation; CI, confidence interval; SRMR, standardized root mean square residual. Changes in fit indices are relative to the immediately preceding, less constrained model.

Scalar longitudinal invariance was supported for SIA and TAPAS, indicating that their scores were measured on a comparable metric across the four waves. Strict invariance was also supported in the additional analysis: the strict SIA model fitted well, χ^2^(761) = 801.603, *p* = 0.149, CFI = 0.999, RMSEA = 0.006, and SRMR = 0.021, as did the strict TAPAS model, χ^2^(761) = 764.696, *p* = 0.456, CFI = 1.000, RMSEA = 0.002, and SRMR = 0.018. Changes from the scalar models were negligible (Supplementary Table S5).

### 3.4 RI-CLPM model comparison and selection

Four nested random-intercept cross-lagged panel models were evaluated to determine whether the autoregressive and cross-lagged parameters could be treated as stable across the three approximately 12-week intervals. The fit statistics for these models are presented in Table 4. The fully unconstrained model, M1, demonstrated good fit, χ^2^(9) = 25.702, *p* = 0.002, CFI = 0.998, TLI = 0.993, RMSEA = 0.033, 90% CI [0.019, 0.049], and SRMR = 0.014.

**TABLE 4:** Fit comparison of alternative random-intercept cross-lagged panel models.

Model	Constraint specification	χ^2^ (*df*)	*p*	CFI	TLI	RMSEA [90% CI]	SRMR	ΔCFI	ΔRMSEA	ΔSRMR	AIC	BIC
M1	Fully unconstrained	25.702 (9)	0.002	0.998	0.993	0.033 [0.019, 0.049]	0.014	–	–	–	80,146.504	80,336.349
M2	Autoregressive paths constrained equal	29.362 (13)	0.006	0.998	0.995	0.027 [0.014, 0.041]	0.015	0.000	-0.006	0.001	80,142.164	80,310.313
M3	Cross-lagged paths constrained equal	28.097 (13)	0.009	0.998	0.996	0.026 [0.013, 0.040]	0.013	0.000	-0.007	-0.001	80,140.899	80,309.048
M4	Autoregressive and cross-lagged paths constrained equal	31.038 (17)	0.020	0.998	0.997	0.022 [0.009, 0.034]	0.014	0.000	-0.011	0.000	80,135.840	80,282.292

CFI, comparative fit index; TLI, Tucker–Lewis index; RMSEA, root mean square error of approximation; CI, confidence interval; SRMR, standardized root mean square residual; AIC, Akaike information criterion; BIC, Bayesian information criterion. Changes in fit indices are relative to the fully unconstrained model, M1. Equality constraints were imposed on the unstandardized coefficients. Lower AIC and BIC values indicate greater relative parsimony.

Constraining only the autoregressive paths to equality over time in M2 or only the cross-lagged paths to equality in M3 did not result in a meaningful deterioration in model fit. The fully equal-lag model, M4, in which both the autoregressive and directional cross-lagged coefficients were constrained to equality across the three intervals, also fitted the data well, χ^2^(17) = 31.038, *p* = 0.020, CFI = 0.998, TLI = 0.997, RMSEA = 0.022, 90% CI [0.009, 0.034], and SRMR = 0.014. Relative to M1, M4 produced no deterioration in CFI or SRMR and a lower RMSEA. It also yielded the lowest Akaike information criterion and Bayesian information criterion among the four models. Accordingly, M4 was retained as the final, more parsimonious RI-CLPM.

The equality restrictions were imposed on the unstandardized lagged coefficients. Consequently, the corresponding standardized coefficients could differ slightly across waves because the variances of the within-person components were not identical at each measurement occasion.

### 3.5 Between- and within-person RI-CLPM results

**FIGURE 3:**
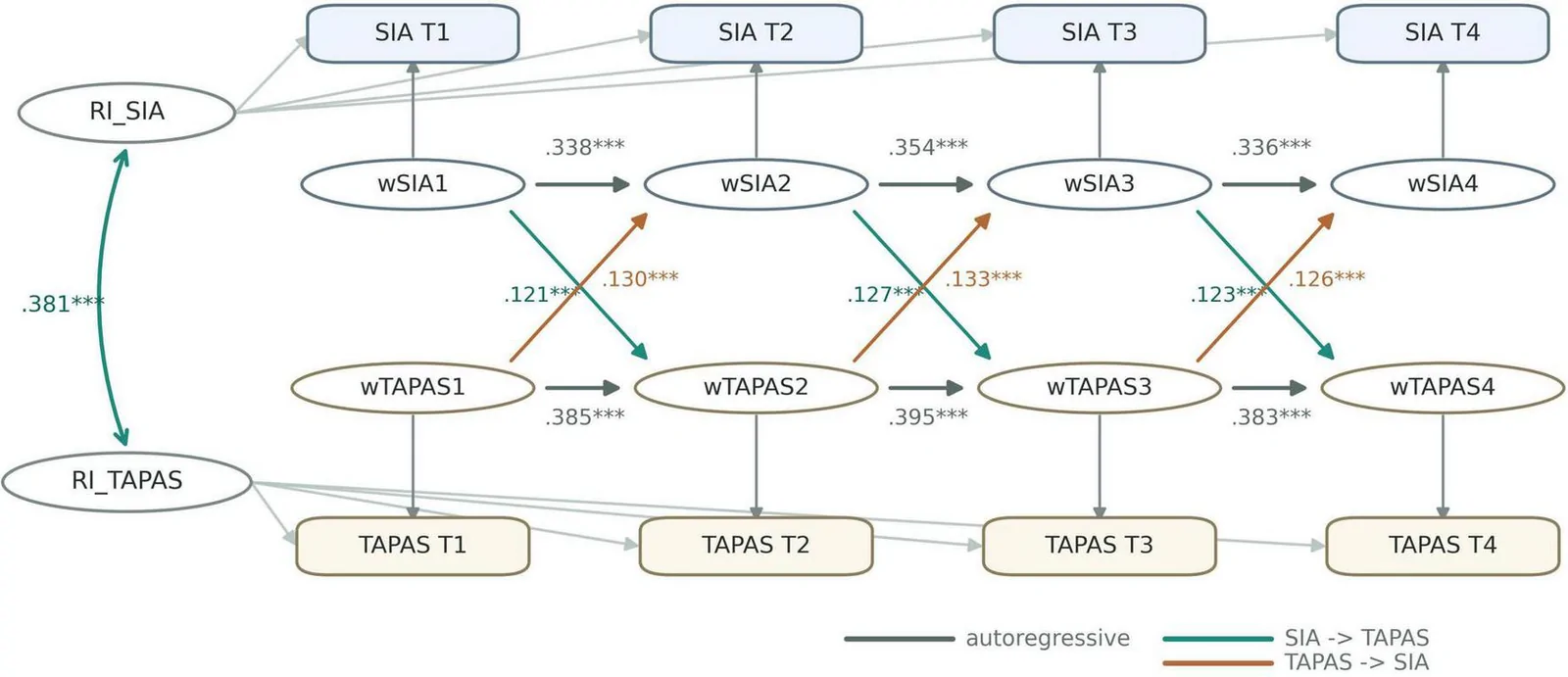
Standardized estimates from the final four-wave random-intercept cross-lagged panel model. SIA = social interaction anxiety; TAPAS = tendency to avoid physical activity and sport; RI_SIA and RI_TAPAS = random intercepts representing stable between-person differences; wSIA and wTAPAS = within-person deviations from individuals’ expected scores; T1–T4 = Time 1–Time 4. Values are STDYX standardized estimates from the final equal-lag model, M4. Equality constraints were imposed on the unstandardized autoregressive and cross-lagged coefficients; therefore, standardized estimates vary slightly across intervals because wave-specific variances differ. Horizontal arrows represent autoregressive paths, and diagonal arrows represent reciprocal cross-lagged paths. The correlation between the random intercepts is shown by the curved double-headed arrow. Within-wave covariances and nonfocal parameters are omitted for visual clarity. ***p < 0.001.

**FIGURE 4:**
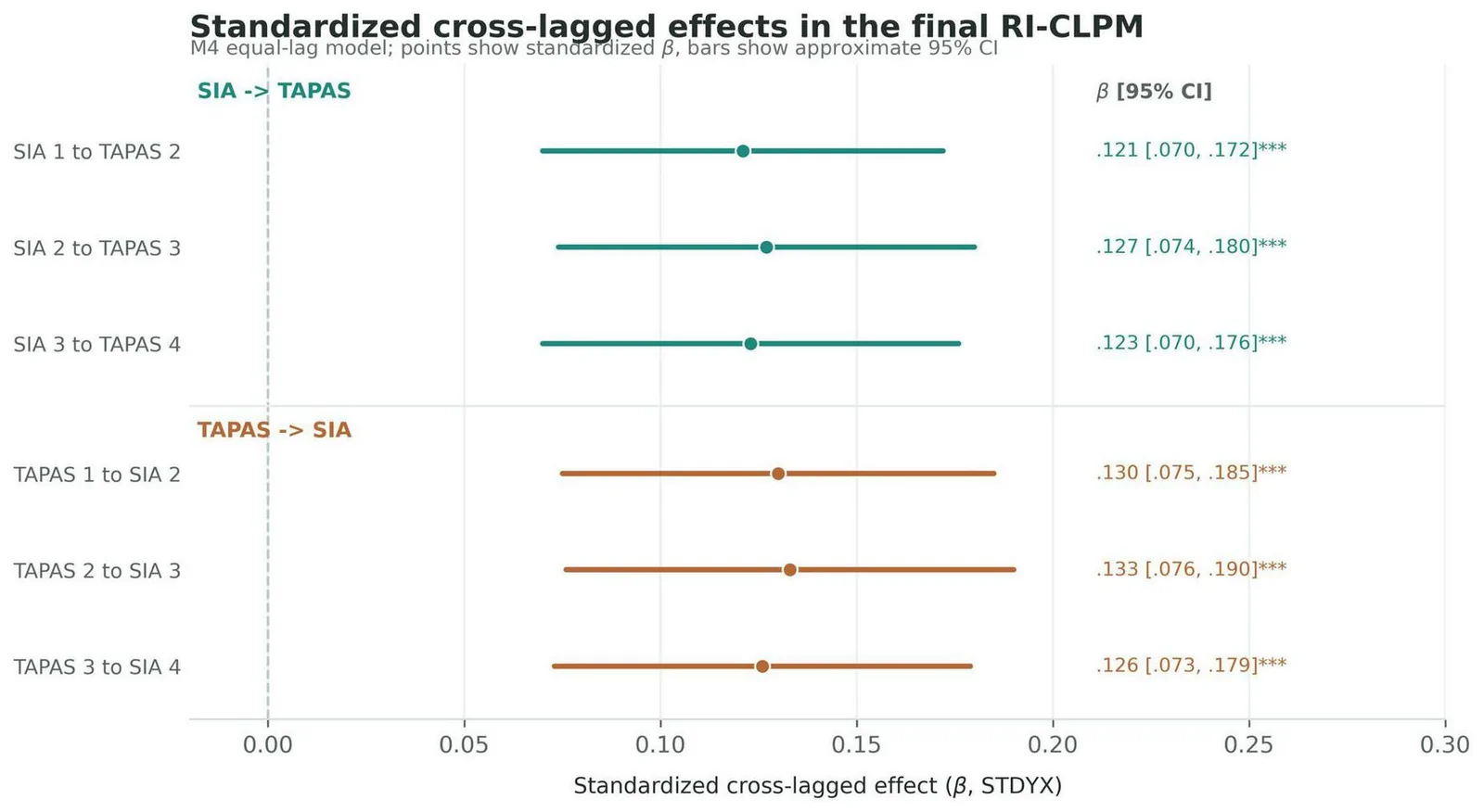
Standardized cross-lagged effects in the final random-intercept cross-lagged panel model. SIA = social interaction anxiety; TAPAS = tendency to avoid physical activity and sport; T1–T4 = Time 1–Time 4. Points represent STDYX standardized cross-lagged coefficients, and horizontal bars represent approximate 95% confidence intervals. The vertical dashed line denotes a null effect of zero. Equality constraints were imposed on the corresponding unstandardized coefficients across the three intervals; small differences among the standardized coefficients result from wave-specific differences in variance. All displayed effects were statistically significant at *p* < 0.001.

The final RI-CLPM estimates are presented in Figure 3, and the standardized cross-lagged effects with their 95% confidence intervals are displayed in Figure 4.

At the between-person level, the random intercepts of SIA and TAPAS were moderately and positively correlated, *r* = 0.381, SE = 0.032, 95% CI [0.318, 0.444], *p* < 0.001. Thus, students who experienced higher average SIA across the four-wave study period also tended to report a stronger average tendency to avoid physical activity and sport. This finding supported H1.

At the within-person level, both constructs demonstrated significant temporal continuity after their stable between-person components had been removed. For SIA, the equality-constrained unstandardized autoregressive coefficient was b = 0.348, SE = 0.036, *p* < 0.001. The corresponding standardized coefficients were β = 0.338, 95% CI [0.279, 0.397], from T1 to T2; β = 0.354, 95% CI [0.280, 0.428], from T2 to T3; and β = 0.336, 95% CI [0.260, 0.412], from T3 to T4. Thus, students reporting higher-than-usual SIA at one wave tended to continue reporting higher-than-usual SIA approximately 12 weeks later.

TAPAS showed a similar but slightly stronger autoregressive pattern. The equality-constrained unstandardized coefficient was b = 0.388, SE = 0.034, *p* < 0.001. The standardized effects were β = 0.385, 95% CI [0.328, 0.442], from T1 to T2; β = 0.395, 95% CI [0.326, 0.464], from T2 to T3; and β = 0.383, 95% CI [0.312, 0.454], from T3 to T4.

Consistent with H2, within-person elevations in SIA significantly predicted subsequent elevations in TAPAS after controlling for prior TAPAS and the reverse cross-lagged pathway. The equality-constrained unstandardized SIA-to-TAPAS coefficient was b = 0.128, SE = 0.028, *p* < 0.001. In standardized terms, the effect was β = 0.121, 95% CI [0.070, 0.172], from T1 SIA to T2 TAPAS; β = 0.127, 95% CI [0.074, 0.180], from T2 SIA to T3 TAPAS; and β = 0.123, 95% CI [0.070, 0.176], from T3 SIA to T4 TAPAS. These findings indicate that when a student experienced more SIA than was typical for that student, the student subsequently reported a stronger-than-usual tendency to avoid physical activity and sport.

The reverse pathway was also consistently significant, supporting H3. The equality-constrained unstandardized TAPAS-to-SIA coefficient was b = 0.127, SE = 0.028, *p* < 0.001. The corresponding standardized effects were β = 0.130, 95% CI [0.075, 0.185], from T1 TAPAS to T2 SIA; β = 0.133, 95% CI [0.076, 0.190], from T2 TAPAS to T3 SIA; and β = 0.126, 95% CI [0.073, 0.179], from T3 TAPAS to T4 SIA. Thus, when a student reported greater exercise-related avoidance than was typical for that student, the student subsequently experienced higher-than-usual SIA.

Overall, the significant cross-lagged paths in both directions supported a reciprocal within-person pattern. Across approximately 12-week intervals, elevations in SIA preceded increases in TAPAS, while elevations in TAPAS also preceded increases in SIA. The similar pattern observed across all three intervals was consistent with the proposed self-reinforcing avoidance process.

### 3.6 Sensitivity analyses

The equal-lag specification remained preferable to the unconstrained model: imposing equality on the autoregressive and directional cross-lagged paths did not worsen fit, Δχ^2^(8) = 5.336, *p* = 0.721, and M4 had lower AIC and BIC values than M1.

The covariate-adjusted RI-CLPM also fitted well, χ^2^(65) = 80.090, *p* = 0.098, CFI = 0.998, TLI = 0.997, RMSEA = 0.012, 90% CI [0.000, 0.020], and SRMR = 0.013. The between-person association remained significant, *r* = 0.387, 95% CI [0.324, 0.450], *p* < 0.001.

Adjustment did not alter the within-person pattern. The SIA-to-TAPAS paths remained positive from T1 to T2, β = 0.122, 95% CI [0.071, 0.173]; T2 to T3, β = 0.128, 95% CI [0.075, 0.181]; and T3 to T4, β = 0.124, 95% CI [0.073, 0.175]. The TAPAS-to-SIA paths were likewise positive from T1 to T2, β = 0.132, 95% CI [0.077, 0.187]; T2 to T3, β = 0.136, 95% CI [0.079, 0.193]; and T3 to T4, β = 0.129, 95% CI [0.076, 0.182]. All adjusted cross-lagged effects were significant at *p* < 0.001.

Attrition analyses identified one significant continuous baseline difference. Students observed at T3 had lower baseline SIA (M = 17.65, SD = 8.79) than those missing at T3 (M = 19.97, SD = 8.79), Welch t(236.71) = −3.42, *p* < 0.001, Hedges g = −0.265, 95% CI [−0.417, −0.113]. In the multivariable model, a one-standard-deviation increase in baseline SIA was associated with lower odds of T3 observation, OR = 0.774, 95% CI [0.662, 0.904], *p* = 0.001. No corresponding association was detected at T2 or T4, and none of the 15 categorical baseline comparisons was significant. These findings identify an observed predictor of wave-specific missingness without confirming MAR.

Item nonresponse was limited. Across the eight scale-by-wave combinations, 869 scores were prorated from nine valid items and 31 from eight valid items; the maximum item-missingness percentage among wave participants was 1.37% (Supplementary Table S4). Requiring all 10 items produced a well-fitting M4, χ^2^(17) = 24.338, *p* = 0.111, CFI = 0.999, TLI = 0.998, RMSEA = 0.016, and SRMR = 0.013. The largest absolute change across the six standardized cross-lagged paths was 0.010, and all paths retained their direction and significance.

The nine-item TAPAS sensitivity analysis also supported the primary findings. The M4 model fitted well, χ^2^(17) = 32.315, *p* = 0.014, CFI = 0.998, TLI = 0.997, RMSEA = 0.023, and SRMR = 0.015. All six cross-lagged paths remained significant in the same direction, with a maximum absolute standardized change of 0.005 (Supplementary Tables S6, S7).

In the Monte Carlo sensitivity analysis, the obtained baseline sample of 1,676 achieved at least 0.80 power in all evaluated scenarios with standardized cross-lagged effects of 0.10 or 0.12. Thresholds for an effect of 0.07 varied with the assumed intraclass correlation and attrition rate, while every 0.05-effect scenario required more than 2,000 baseline participants. Supplementary Table S8 summarizes the scenario ranges.

## 4 Discussion

### 4.1 Principal findings: a reciprocal avoidance process

The study identified two complementary patterns. Students with higher average SIA across the academic year also reported higher average TAPAS, and wave-specific elevations in either construct predicted elevations in the other approximately 12 weeks later. The reciprocal within-person paths were reproduced across all three intervals, remained after adjustment for baseline characteristics, and were robust to alternative TAPAS scoring and complete-item requirements. The findings are consistent with the proposed avoidance-trap heuristic: social-evaluative anxiety may make exercise settings more avoidable, while exercise-related avoidance may preserve conditions under which anxiety remains elevated.

The central theoretical contribution lies in the coexistence of stable between-person covariation and dynamic within-person reciprocity. The random-intercept correlation describes enduring differences between students, which may reflect body-related concerns, sensitivity to scrutiny, or prior stigmatizing experiences. The cross-lagged paths address fluctuations within the same student and show that the association was not limited to chronic differences between people. The results therefore support a multilevel account in which stable vulnerability and time-specific reinforcement can operate together (Curran and Bauer, 2011; Usami et al., 2019).

The standardized cross-lagged coefficients were modest (β≈ 0.12–0.13). Direct comparison with coefficients from other RI-CLPM studies is limited because estimates depend on the measurement interval, reliability, stability, and the proportion of variance separated into within- and between-person components. Their relevance here rests on their consistency across three intervals after autoregression, the competing pathway, and stable between-person variance were modeled. The similar estimates in the two directions do not establish equal strength, because the model constrained each direction across time without constraining the two directions to one another. The results describe a medium-term academic-year process and do not imply the same pattern over days or years. Their practical importance depends on repetition and context rather than a universal numerical benchmark (Götz et al., 2022).

### 4.2 Why social interaction anxiety may increase subsequent avoidance of physical activity and sport

The SIA-to-TAPAS pathway is consistent with physical activity and sport becoming extensions of a broader social-evaluative threat system. When SIA rises, students may attend more closely to signs of observation and overestimate the interpersonal consequences of appearing awkward or inadequate. Exercise settings can expose physique, coordination, competence, sweating, fatigue, and interaction with unfamiliar peers, making feared self-attributes especially salient (Moscovitch, 2009). Gyms, group fitness classes, team sports, and compulsory physical education may therefore feel threatening for reasons that extend beyond exercise itself.

This interpretation aligns with evidence linking social physique anxiety, weight stigma, and exercise avoidance (Brunet and Sabiston, 2009; Thedinga et al., 2021). The present findings extend that literature by showing that changes in general interaction anxiety preceded changes in exercise-related avoidance within the same student. The outcome concerns avoidance tendency rather than minutes of physical activity; objective participation may still be shaped by access, injury, academic demands, and mandatory courses.

### 4.3 Why avoidance of physical activity and sport may increase subsequent social interaction anxiety

The reverse TAPAS-to-SIA pathway is consistent with two closely related maintenance processes. Avoidance can bring immediate relief, reinforcing future withdrawal, and it can prevent threat expectations from being revised through direct experience (Craske et al., 2014; Krypotos et al., 2015). Avoiding exercise settings may therefore remove opportunities for manageable visibility, benign peer feedback, physical mastery, and informal social contact.

Repeated withdrawal from these settings could preserve beliefs that participation would be humiliating or socially costly and may narrow opportunities for rewarding interaction. TAPAS may also reflect a broader avoidance orientation that was not measured directly. The study cannot distinguish these mechanisms, and an avoided exercise opportunity should not be interpreted as causing later anxiety. The prospective association nevertheless indicates that exercise-related avoidance carried information about subsequent SIA beyond prior SIA, stable individual differences, and the simultaneous anxiety-to-avoidance pathway.

### 4.4 Theoretical implications

The distinction between stable co-occurrence and within-person change is the main theoretical advance. Accounts of SIA and TAPAS should explain why some students remain persistently higher than their peers and why either construct rises above a student’s usual level at a particular time. Treating these as separate estimands prevents enduring differences from being mistaken for change processes and gives greater precision to claims about reciprocity.

The findings also broaden the contexts considered in models of social anxiety. Physical activity and sport combine interpersonal evaluation with bodily visibility, allowing exercise-related avoidance to be understood as a context-specific expression of social-evaluative concerns. TAPAS may therefore have relevance beyond its association with lower activity participation and may form part of a broader anxiety-maintenance process. The evidence supports this interpretation as a hypothesis for further testing rather than as a settled causal mechanism.

The process is time-scale specific. The model supports reciprocal prospective associations over approximately 12 weeks across one academic year. Intensive longitudinal designs are needed to determine whether SIA affects avoidance within hours or days and whether repeated short-term episodes accumulate into the medium-term pattern observed here.

### 4.5 Practical implications

The practical implications are best treated as candidate directions for intervention development and evaluation. Campus programs may consider assessing social-evaluative concerns when students report difficulty participating in physical activity. Exercise settings could offer a wider range of individual, small-group, beginner, skill-matched, noncompetitive, and relatively private options. Emphasizing enjoyment, function, belonging, and personal choice may also reduce avoidable appearance- and performance-focused pressure.

For students with elevated SIA and TAPAS, intervention studies could evaluate whether voluntary, individually paced participation in manageable exercise settings helps students test feared predictions while preserving autonomy. Psychological support and physical-activity programming may be more useful when coordinated than when either component is offered in isolation. These possibilities derive from the observed reciprocal pattern and require experimental evaluation before conclusions about effectiveness can be drawn.

The findings also caution against assuming that greater provision alone will remove barriers. An environment can be physically accessible while remaining socially threatening. Collaboration among counseling services, physical education staff, and recreational-sport providers may help identify modifiable features of exercise settings, but the present study does not show that changing either SIA or TAPAS will necessarily change the other.

## 5 Limitations and future directions

The findings should be interpreted within several limitations. The observational RI-CLPM separated stable between-person differences from time-specific deviations, yet time-varying confounders could still generate cross-lagged associations. Stigmatizing experiences, injury, depressive symptoms, examination stress, body dissatisfaction, residential changes, or appearance-focused social media exposure may affect both constructs between waves. Repeated measurement of such factors and experimental or quasi-experimental designs are needed for stronger causal tests (Rohrer and Murayama, 2023).

Both constructs were assessed by self-report within the same survey. Measurement invariance supports longitudinal comparability but does not remove shared-method variance or mood-congruent reporting. TAPAS measures avoidance tendency rather than observed avoidance or physical-activity volume; future studies should combine it with attendance records, accelerometry, or brief opportunity-based diaries. SIAS-10 scores also represent a continuous symptom domain and do not establish a diagnosis of social anxiety disorder.

The approximately 12-week interval was suited to medium-term academic-year associations but may miss shorter mechanisms. The T2–T3 interval crossed the winter recess, when social contexts and access to campus facilities may have changed. Although the equal-lag model fitted well, daily or weekly measurement bursts and continuous-time models could clarify how the association varies across shorter and longer lags.

The selected RI-CLPM is one plausible representation of the data. It assumes common lagged effects across participants and does not model individual differences in growth. Alternative longitudinal models address different estimands and may yield different conclusions, so future work should compare RI-CLPM results with growth-based and heterogeneous-effect specifications (Orth et al., 2021; Usami et al., 2019).

Participants were recruited through classroom-based convenience sampling at nine universities in one Chinese province. The sample should not be treated as representative of all Chinese university students. Norms concerning body visibility, peer evaluation, compulsory physical education, and acceptable forms of exercise participation may differ across regions, institutions, and national contexts. No information was collected from students who declined or did not return a baseline questionnaire, preventing evaluation of respondent–nonrespondent differences.

Wave-specific missingness increased from 9.4% at T2 to 17.0% at T4. Higher baseline SIA predicted nonobservation at T3, while other examined baseline variables did not show consistent associations with participation. FIML incorporated all available data and can accommodate missingness related to observed model variables under MAR, but the analyses cannot confirm MAR or rule out unmeasured reasons for nonresponse. Recording reasons for absence and adding auxiliary predictors would strengthen future missing-data sensitivity analyses (Enders and Bandalos, 2001; Graham, 2009).

The covariate analysis addressed stable demographic differences and did not test whether within-person pathways varied across sex, weight status, body dissatisfaction, stigma exposure, or exercise context. Studies designed for moderation analyses could identify students and settings for which the reciprocal process is strongest.

## 6 Conclusion

Across four waves, SIA and TAPAS showed both stable between-person covariation and reciprocal within-person prospective associations. The findings are consistent with a medium-term reciprocal avoidance process, while the observational design precludes causal conclusions. Experimental and intensive longitudinal studies are needed to test the proposed mechanisms and candidate intervention targets. More broadly, separating stable between-person co-occurrence from dynamic within-person processes clarifies how psychological symptoms and health-related avoidance may become coupled over time.

## Data Availability

The original contributions presented in the study are included in the article/Supplementary material, further inquiries can be directed to the corresponding author.
