# Supplementary material for: An avoidance trap? Within-person dynamics between social interaction anxiety and the tendency to avoid physical activity and sport among Chinese university students: a four-wave random-intercept cross-lagged panel study
Source: Front Psychol. 2026 Aug 21;17:1914632. doi: 10.3389/fpsyg.2026.1914632 (PMC13541471; doi:10.3389/fpsyg.2026.1914632)
Supplement: Supplementary file 1 [file Supplementary_file_1.docx]

**MANUSCRIPT ID 1914632**

**Supplementary Analysis**

Attrition, item missingness, longitudinal invariance, scoring sensitivity, and Monte Carlo analyses

Software: R 4.5.3 and Mplus 8.3

# Supplementary Methods

## Attrition and MAR-plausibility analyses

Observation was modeled separately at T2, T3, and T4 using the same occasion-specific follow-up indicators as the participant-flow analysis. These indicators reproduced the reported observed counts of 1,519 at T2, 1,488 at T3, and 1,391 at T4. Scale-specific item nonresponse was examined separately and did not redefine wave-level observation. Baseline SIA, TAPAS, age, sex, year of study, pre-university residence, and BMI category were compared between observed and missing participants. Multivariable logistic models predicted observation from standardized baseline SIA and TAPAS plus sex, year of study, residence, and BMI category. These analyses assess observed predictors of missingness; they cannot confirm MAR or exclude unmeasured causes of nonresponse.

## Item nonresponse and scoring sensitivity

A prespecified study-specific rule required at least eight valid responses for each 10-item scale. Scores based on eight or nine responses were prorated to the original total-score metric by multiplying the available-item mean by 10. Fewer than eight valid responses yielded a missing scale score. Item completeness was summarized by scale and wave, separating within-wave item nonresponse from whole-wave nonparticipation. A complete-item sensitivity analysis required all 10 items while retaining the baseline cohort and FIML estimation.

## Strict longitudinal invariance

Following the configural, metric, and scalar models, strict models additionally constrained corresponding residual variances across waves. Same-item residual covariances and all other identification choices were retained. Model comparisons used absolute and incremental fit indices together with the chi-square difference test.

## Nine-item TAPAS sensitivity

The nine-item sensitivity score omitted Item 10 at all waves: “I would prefer to participate in physical activity in a more private setting.” Reliability, longitudinal invariance, M1–M4 RI-CLPMs, and the covariate-adjusted M4 were re-estimated using the same cohort and structural specification as the 10-item analysis. For the nine-item score, at least eight valid responses were required; scores based on eight responses were prorated to the nine-item metric.

## Monte Carlo sensitivity of sample-size assumptions

A Monte Carlo sensitivity analysis evaluated sample-size adequacy across a range of population assumptions. The analysis crossed standardized focal cross-lagged effects of .05, .07, .10, and .12 with intraclass correlations of .30, .50, and .60 and cumulative T4 attrition rates of 15%, 20%, and 25%. Each focal direction was evaluated separately, producing 72 directional scenarios. Other population parameters were held constant: random-intercept correlation = .47, within-wave correlation = .20, SIA autoregression = .36, TAPAS autoregression = .40, and time-invariant effects across four waves. Wave-specific MCAR missingness increased linearly to the selected T4 rate. At least 5,000 replications were used per scenario. Baseline sample sizes from 200 to 2,000 were searched and locally refined to identify the approximate threshold for 80% power. The grid evaluated the sensitivity of the sample-size conclusion and was not used to determine recruitment.

# Supplementary Results

## Supplementary Table S1. Occasion-specific observation and wave-level missingness

| Wave | Observed n | Observed % of T1 | Missing n | Missing % of T1 |
| --- | --- | --- | --- | --- |
| T1 | 1,676 | 100.0% | 0 | 0.0% |
| T2 | 1,519 | 90.6% | 157 | 9.4% |
| T3 | 1,488 | 88.8% | 188 | 11.2% |
| T4 | 1,391 | 83.0% | 285 | 17.0% |

***Note.*** *Percentages were calculated relative to the T1 analytic cohort (N = 1,676). Observation was occasion-specific. Participants missing at one wave remained eligible to participate later; wave-specific missing groups could therefore overlap.*

## Supplementary Table S2. Significant continuous baseline difference in the attrition analyses

| Wave | Variable | Observed M (SD) | Missing M (SD) | t | df | p | Hedges g | 95% CI |
| --- | --- | --- | --- | --- | --- | --- | --- | --- |
| T3 | Baseline SIA | 17.65 (8.79) | 19.97 (8.79) | -3.42 | 236.71 | < .001 | -.265 | [-.417, -.113] |

***Note.*** *Welch tests and Hedges g are reported. This was the only significant continuous comparison. None of the 15 categorical baseline comparisons was significant.*

## Supplementary Table S3. Focal predictors from multivariable wave-observation models

| Wave | Predictor | B | SE | OR | 95% CI | p |
| --- | --- | --- | --- | --- | --- | --- |
| T2 | Baseline SIA (z) | -.112 | .085 | .894 | [.756, 1.056] | .187 |
| T2 | Baseline TAPAS (z) | -.106 | .087 | .899 | [.758, 1.067] | .223 |
| T3 | Baseline SIA (z) | -.257 | .080 | .774 | [.662, .904] | .001 |
| T3 | Baseline TAPAS (z) | -.043 | .080 | .958 | [.818, 1.121] | .591 |
| T4 | Baseline SIA (z) | -.042 | .067 | .959 | [.842, 1.093] | .528 |
| T4 | Baseline TAPAS (z) | -.090 | .067 | .914 | [.801, 1.043] | .181 |

***Note.*** *Outcome = observed at the specified wave. Models also included sex, year of study, pre-university residence, and BMI category.*

## Supplementary Table S4. Frequency of direct and prorated scale scores

| Scale | Wave | 10 valid | 9 valid | 8 valid | Prorated total |
| --- | --- | --- | --- | --- | --- |
| SIA | T1 | 1,569 | 106 | 1 | 107 |
| SIA | T2 | 1,392 | 124 | 3 | 127 |
| SIA | T3 | 1,381 | 99 | 7 | 106 |
| SIA | T4 | 1,285 | 101 | 4 | 105 |
| TAPAS | T1 | 1,556 | 114 | 5 | 119 |
| TAPAS | T2 | 1,389 | 126 | 4 | 130 |
| TAPAS | T3 | 1,382 | 102 | 4 | 106 |
| TAPAS | T4 | 1,290 | 97 | 3 | 100 |

Note. Scores based on nine or eight valid items were prorated. Across all scale-wave combinations, 869 scores used nine items and 31 used eight items. Maximum item missingness among wave participants was 1.37%. One valid T1 TAPAS total score in the analysis dataset did not have a corresponding item-level audit record and is therefore not represented in the T1 item-completeness counts; this did not affect any scale-score or model analysis.

## Supplementary Table S5. Strict longitudinal invariance

| Scale | χ² (df) | p | CFI | TLI | RMSEA [90% CI] | SRMR | Δχ² (df) | Δp |
| --- | --- | --- | --- | --- | --- | --- | --- | --- |
| SIA | 801.603 (761) | .149 | .999 | .999 | .006 [.000, .009] | .021 | 33.791 (30) | .289 |
| TAPAS | 764.696 (761) | .456 | 1.000 | 1.000 | .002 [.000, .008] | .018 | 23.639 (30) | .788 |

***Note.*** *Difference statistics compare strict with scalar models. Strict invariance was supported for both constructs.*

## Supplementary Table S6. Nine-item TAPAS descriptives and reliability

| Wave | n | M | SD | α | ω (polychoric) | r with 10-item |
| --- | --- | --- | --- | --- | --- | --- |
| T1 | 1,672 | 26.75 | 8.13 | .903 | .917 | .995 |
| T2 | 1,515 | 26.91 | 8.21 | .907 | .920 | .995 |
| T3 | 1,484 | 27.27 | 8.16 | .906 | .920 | .995 |
| T4 | 1,387 | 27.50 | 8.09 | .904 | .918 | .995 |

***Note.*** *Omega is a one-factor polychoric estimate. Correlations with the 10-item score are pairwise.*

The nine-item M4 fitted well, χ²(17) = 32.315, p = .014, CFI = .998, TLI = .997, RMSEA = .023, and SRMR = .015. All six cross-lagged paths remained significant and in the same direction; the maximum absolute standardized change was .005. The primary reciprocal pattern therefore remained substantively unchanged.

## Supplementary Table S7. Primary and scoring-sensitivity M4 model fit

| Analysis | χ² (df) | p | CFI | TLI | RMSEA [90% CI] | SRMR | Max \|Δβ\| |
| --- | --- | --- | --- | --- | --- | --- | --- |
| Primary 10-item, ≥8/10 | 31.038 (17) | .020 | .998 | .997 | .022 [.009, .034] | .014 | Reference |
| Nine-item TAPAS | 32.315 (17) | .014 | .998 | .997 | .023 [.010, .035] | .015 | .005 |
| Complete-item 10/10 | 24.338 (17) | .111 | .999 | .998 | .016 [.000, .029] | .013 | .010 |

***Note.*** *Max |Δβ| is the maximum absolute change across the six standardized cross-lagged paths relative to the primary model. All six paths retained their direction and statistical significance in both sensitivity analyses.*

## Supplementary Table S8. Aggregate 80% power thresholds across ICC and attrition scenarios

| Focal direction | Effect | Resolved N range | Scenarios >2,000 | Scenarios below 1,676 |
| --- | --- | --- | --- | --- |
| SIA → TAPAS | .05 | >2,000 | 9 | 0 |
| SIA → TAPAS | .07 | 1,360–1,910 | 0 | 5 |
| SIA → TAPAS | .10 | 640–930 | 0 | 9 |
| SIA → TAPAS | .12 | 460–660 | 0 | 9 |
| TAPAS → SIA | .05 | >2,000 | 9 | 0 |
| TAPAS → SIA | .07 | 1,490–1,980 | 1 | 2 |
| TAPAS → SIA | .10 | 720–1,050 | 0 | 9 |
| TAPAS → SIA | .12 | 510–740 | 0 | 9 |

***Note.*** *Each effect-by-direction row summarizes nine combinations of ICC (.30, .50, .60) and cumulative T4 attrition (15%, 20%, 25%). The obtained baseline sample of 1,676 exceeded the 80% threshold in every evaluated .10 and .12 scenario. Adequacy for a .07 effect depended on the assumptions; every .05 scenario required more than 2,000 baseline participants.*
